# CpG 1018 augments mRNA vaccine-induced anti-tumor immunity by potentiating CD8+ T cell responses

**DOI:** 10.1016/j.omton.2026.201230

**Published:** 2026-05-09

**Authors:** Yibo Li, Jayachandra Reddy Nakkala, Labone Akter, Xinliang Kang, Yuna Song, Elise VanLuinen, Xinyuan Chen

**Affiliations:** 1 Biomedical & Pharmaceutical Sciences, College of Pharmacy, University of Rhode Island, 7 Greenhouse Road, Avedisian Hall, Room 480, Kingston, RI 02881, USA

**Keywords:** MT:, mRNA vaccine, cancer vaccine, tumor vaccine, neoantigen mRNA vaccine, personalized neoantigen mRNA vaccine, CpG 1018, immunotherapy, melanoma, neoantigen, NK cells, vaccine adjuvant

## Abstract

Personalized neoantigen mRNA vaccines showed good efficacy in treating metastatic melanoma, pancreatic cancer, and breast cancer in early phase clinical trials. Strategies are needed to further enhance neoantigen mRNA vaccine-induced anti-tumor immunity. This study explored conventional adjuvant co-administration to enhance mRNA vaccine-induced anti-tumor immunity. We found a clinically used CpG 1018 adjuvant was highly effective to enhance ovalbumin (OVA) and neoantigen mRNA-induced T cell responses and anti-tumor immunity in B16F10-OVA melanoma models. Mechanistic studies found CpG 1018 mainly enhanced dendritic cell maturation and local cytokine/chemokine release but not mRNA translation. mRNA vaccine in the presence of CpG 1018 induced significantly higher levels of Granzyme B, IFNγ, and TNFα-secreting CD8+ T cells as compared to mRNA vaccine alone. We further found that potent anti-tumor immunity was associated with increased tumor-infiltration of CD8+ T cells and positively correlated with tumor-infiltrating CD8+ to CD4+ T cell ratios. Cell depletion studies found CD8+ T cells rather than CD4+ T cells or NK cells played crucial roles in CpG 1018-augmented mRNA vaccine efficacy. CpG 1018-adjuvanted mRNA vaccine induced transient body weight loss (<6%) with an overall good safety. Our data warrant further investigation of CpG 1018-adjuvanted neoantigen mRNA vaccine for potentially better tumor therapy.

## Graphical abstract

**Figure:**
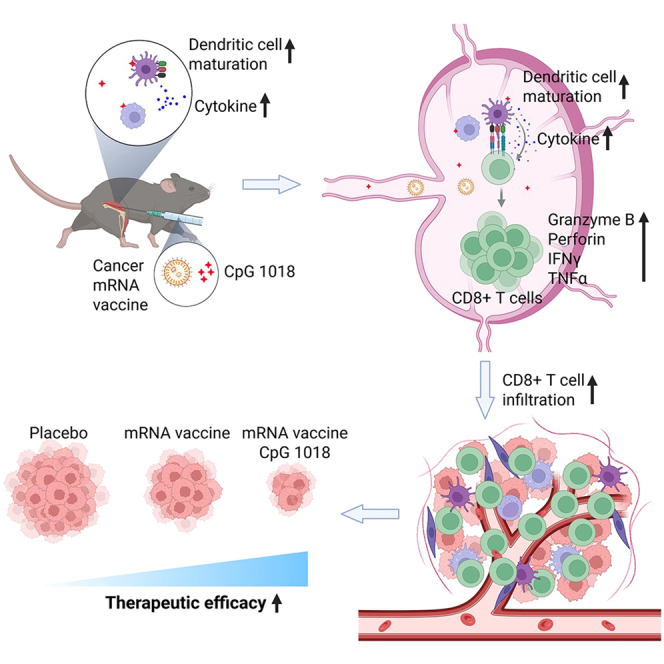

## Teaser

Strategies are sought to enhance personalized cancer mRNA vaccine efficacy to achieve long-term remission. A clinically used CpG 1018 adjuvant shows high potency to potentiate mRNA vaccine-induced T cell responses and anti-tumor immunity in low immunogenic B16F10 melanoma models.

## Introduction

mRNA evolves as a novel vaccine platform and was successfully used for the rapid development of coronavirus disease 2019 (COVID-19) vaccines. mRNA vaccine platforms have since been actively explored in cancer vaccine development. Neoantigen (NeoAg)-based mRNA vaccines against melanoma, pancreatic ductal adenocarcinoma (PDAC), and triple-negative breast cancer (TNBC) generated promising results in recent early phase clinical trials.^1^^,^^2^^,^^3^^,^^4^ Personalized NeoAg mRNA vaccine (mRNA-4157/V940) in combination with immune checkpoint inhibitor (ICI, pembrolizumab) elicited 44% higher rates of recurrence-free survival (RFS) in resected stage 3/4 melanoma patients as compared to pembrolizumab alone in a randomized phase 2b KEYNOTE-942 trial.^2^^,^^3^ Personalized NeoAg mRNA vaccine (autogene cevumeran) in combination with ICI atezolizumab and chemotherapy (mFOLFIRINOX) elicited longer median RFS in vaccine responders (not reached) than vaccine non-responders (13.4 months) in treatment of PDAC.^1^ Individualized NeoAg mRNA vaccines permitted RFS beyond 5 years in 10 out of 14 TNBC patients.^4^ One TNBC patient remained relapse-free until death from unknown causes.^4^ Three TNBC patients relapsed with one patient having the lowest T cell responses and the other two patients with metastases showing differential mutations from the primary tumor or experiencing nearly complete loss of MHC class I expression.^4^ In these trials, patients underwent tumor resection and next-generation sequencing to predict and rank NeoAgs by immunogenicity.^1^^,^^2^^,^^3^^,^^4^ NeoAg mRNA was then synthesized and encapsulated into lipid nanoparticles (LNPs) or lipoplex nanoparticles for use as cancer vaccines.^1^^,^^2^^,^^3^^,^^4^ It took 6–9 weeks in the melanoma and PDAC trials and an average of about 10 weeks in the TNBC trial to produce NeoAg mRNA vaccines after tumor resection.^1^^,^^2^^,^^3^^,^^4^ The significant extension of survival in early phase trials enrolling less than 20 patients in each treatment arm hinted the high potency of NeoAg mRNA vaccines in tumor therapy.

Highly immunogenic mRNA vaccines can be ascribed to various factors. Structural components, such as 5′ capping, 5′- and 3′-untranslated regions (UTRs), open reading frame (ORF), and 3′ poly(A) tail, have been optimized to achieve efficient mRNA translation *in vivo*.^5^ Antigenic mRNA are often packaged into LNPs or lipoplex nanoparticles to enable efficient mRNA encapsulation, host cell uptake, and cytosol release.^5^ The above PDAC and TNBC trials used non-nucleotide-modified uridine mRNA to strongly stimulate toll-like receptor (TLR)-mediated, type 1 interferon (IFN) responses to expand antigen-specific T cell responses,^1^^,^^4^ while the melanoma trial used pseudouridine-modified mRNA to minimize innate immune activation and achieve more efficient mRNA translation.^2^ Recent studies found mRNA vaccines (pseudouridine-modified mRNA or packaging LNPs) can activate innate immune signaling pathways (e.g., melanoma differentiation-associated gene 5 (*MDA-5*), TLR4) and possess inherent adjuvant effects to boost mRNA vaccination.^6^^,^^7^^,^^8^^,^^9^

Though highly immunogenic, a longer follow-up study of the PDAC trial found two patients with relatively low levels of NeoAg-specific T cells recurred.^10^ The TNBC trail found one patient with the weakest vaccine-induced immune responses relapsed.^4^ These observations indicate the importance of inducing potent NeoAg-specific T cell responses. Additionally, long-term remission demands high-level and long-lasting immunity. Strategies are in need to further enhance mRNA vaccine-induced T cell responses and anti-tumor immunity. Structural components are under further optimization to increase mRNA stability and translational efficiency.^11^ Due to the unique mRNA platform, non-conventional adjuvant approaches have been explored to enhance mRNA vaccine-induced T cell responses that include co-expression of TLR5 agonist flagellin,^12^ co-expression of innate immune sensors (e.g., stimulator of interferon genes [*STING*]),^13^^,^^14^ and replacement of ionizable lipids with TLR7/8-activating adjuvant lipids.^15^ These studies support enhancing mRNA vaccine-induced innate immune responses to bridge more potent T cell responses and anti-tumor immunity particularly for modified mRNA vaccines with relatively weak ability to activate innate immune systems.

Supplementation of mRNA vaccines with traditional adjuvants presents a convenient approach to enhance their efficacy. Considering antigenic mRNA need to be translated in host cells to elicit antigen-specific adaptive immunity, traditional adjuvants that solely enhance protein/subunit antigen uptake may not be effective to boost mRNA vaccination. Adjuvant-induced innate immune responses may also hamper mRNA translation.^16^ The overall impact of adjuvant incorporation on mRNA translation, innate immune system activation, and antigen-specific T cell responses needs to be thoroughly explored to identify the optimal one(s) to boost mRNA vaccine-induced anti-tumor immunity.

CpG oligonucleotides (ODNs) activate TLR9 to potentiate helper T type 1 (Th1) and cytotoxic T lymphocyte (CTL) responses and have been actively explored to enhance protein/peptide-based cancer vaccine efficacy.^17^^,^^18^^,^^19^ CpG 1018 remains the only FDA-approved CpG ODN and is approved to enhance hepatitis B vaccine (HEPLISAV-B) efficacy.^20^ CpG 1018 belongs to class B CpG, strongly activating B cell and nuclear factor kappa B (NF-κB) signaling while weakly activating plasmacytoid DCs (pDCs) and type 1 IFN responses. Different from conventional CpG, which is often species-specific,^21^ CpG 1018 is effective to stimulate TLR9 of rodents, non-human primates, and humans.^20^ The potential of CpG 1018 to enhance mRNA vaccine-induced T cell responses and anti-tumor immunity remains largely unexplored. This study evaluated the potential of CpG 1018 and several widely used chemical adjuvants (e.g., Alum, MF59-like AddaVax, cyclic guanosine monophosphate-adenosine monophosphate [cGAMP]) to enhance model antigen ovalbumin (OVA) and NeoAg mRNA (pseudouridine modified)-induced T cell responses and anti-tumor immunity in highly aggressive, poorly immunogenic (cold) B16F10 melanoma models. We found CpG 1018 remained the only one that vigorously enhanced OVA and NeoAg mRNA-induced CTL responses and anti-tumor immunity due to significantly augmented local innate immunity (e.g., cytokine release, DC maturation). We further found CD8^+^ T cells but not natural killer (NK) cells or CD4^+^ T cells played crucial roles in potent anti-tumor immunity elicited in the presence of CpG 1018.

## Results

### CpG 1018 increases OVA mRNA-induced anti-tumor immunity in preventive B16F10-OVA melanoma models

We first used OVA mRNA and OVA-expressing B16F10 (B16F10-OVA) melanoma to explore the ability of CpG 1018 to enhance OVA mRNA-induced anti-tumor immunity in preventive models. OVA mRNA was encapsulated in LNPs with 95% encapsulation efficiency (EE). LNP (OVA mRNA) had an average diameter of 96.6 nm with good particle uniformity (PDI: 0.119, Figure S1).

**Figure 1:**
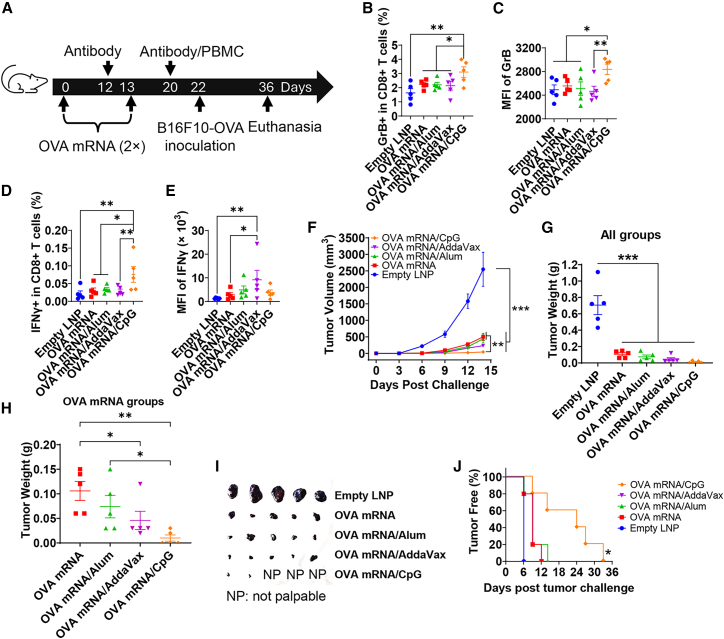
CpG 1018 enhances OVA mRNA-induced anti-tumor immunity in preventive B16F10-OVA models (A) Schematic illustration of experimental design in preventive B16F10-OVA tumor models. (B–E) Cellular immune responses were analyzed 1 week after boost. PBMCs were stimulated with OVA overnight followed by intracellular cytokine staining and flow cytometry analysis. Cells were first gated based on SSC and FSC and then CD4 and CD8 (Figure S2A). CD4^+^ and CD8^+^ T cells were then gated based on GrB and IFNγ (Figure S2A). Percentages of GrB^+^ and IFNγ^+^ cells in CD8^+^ T cells were shown in (B) and (D), respectively. MFI of GrB and IFNγ in GrB^+^CD8^+^ and IFNγ^+^CD8^+^ T cells was shown in (C) and (E), respectively. (F–G) Mice were challenged with 10^6^ OVA-expressing B16F10-OVA melanoma cells 3 weeks after boost. Tumor growth rate was shown in (F). Mice with measurable tumor growth were euthanized 2 weeks after challenge. Tumors were dissected and weighed. Tumor weights of all groups were shown in (G). Tumor weights of mRNA vaccine groups were shown in (H). Tumor images were shown in (I). Percentages of tumor-free mice were shown in (J). One-way ANOVA with Fisher’s LSD test was used to compare differences between groups in (B–E), and (G–H). Two-way ANOVA with Dunnett’s multiple comparisons test was used to compare differences between groups in (F). Logrank (Mantel-Cox) test was used to compare tumor-free percentage between groups (vs. OVA mRNA) in (J). *n* = 5; ∗*p* < 0.05; ∗∗*p* < 0.01; ∗∗∗*p* < 0.001. Experiments were repeated once with similar results.

C57BL/6 mice were prime/boost immunized with OVA mRNA alone (LNP omitted hereafter for simplicity) or admixed with CpG 1018, AddaVax, or Alum, or immunized with empty LNP. AddaVax and Alum adjuvants, mainly enhancing protein/subunit vaccine-induced antibody responses,^22^^,^^23^ were included for comparison. The overall experimental approach was shown in Figure 1A. Cellular immune responses were evaluated 1 week after boosting. CpG 1018 significantly increased OVA mRNA-induced Granzyme B (GrB)^+^ cells in CD8^+^ T cells (Figure 1B) and mean fluorescence intensity (MFI) of GrB in GrB^+^CD8^+^ T cells (Figure 1C). CpG 1018 also significantly increased IFNγ^+^ cells in CD8^+^ T cells (Figure 1D) but not MFI of IFNγ in IFNγ^+^CD8^+^ T cells (Figure 1E). Percentages of GrB^+^ and IFNγ^+^ cells in CD4^+^ T cells were also significantly increased in OVA mRNA/CpG group (Figures S3A and S3C). Interestingly, MFI of GrB and IFNγ showed no significant difference among groups (Figures S3B and S3D).

Humoral immune responses were also monitored. After priming, OVA mRNA induced significant OVA-specific IgG and subtype IgG1 and IgG2c titers (Figures S4A–S4C). Interestingly, incorporation of CpG 1018 significantly reduced OVA mRNA-induced IgG and subtype IgG2c titers and completely inhibited IgG1 antibody production (Figures S4A–S4C). Incorporation of Alum did not significantly affect IgG, subtype IgG1, or IgG2c titers, while incorporation of AddaVax slightly reduced OVA mRNA-induced IgG and subtype IgG2c titers (Figures S4A–S4C). The trend slightly changed after boosting. Incorporation of adjuvants did not significantly affect OVA mRNA-induced IgG, subtype IgG1 or IgG2c antibody responses except AddaVax, which significantly increased OVA mRNA-induced IgG1 antibody responses (Figures S4D–S4F). The significant inhibition of OVA-specific IgG1 antibody responses in the presence of CpG 1018 adjuvant and the more significant inhibition of OVA-specific IgG antibody responses after priming but not after boosting remain to be explored. Overall, these results indicate the minimal effects of chosen adjuvants to enhance OVA mRNA-induced antibody responses. We also monitored the safety of CpG 1018-adjuvanted OVA mRNA vaccination in boost immunization. All immunizations including the empty LNP induced transient weight loss (<5%) (Figure S5A).

The above immunized mice were challenged with B16F10-OVA melanoma. OVA mRNA significantly retarded tumor growth as compared to empty LNP (Figure 1F). Incorporation of CpG 1018 most significantly retarded tumor growth followed by AddaVax, while incorporation of Alum showed no significant impact on tumor growth (Figure 1F). Tumors were harvested on day 14 and weighed. Tumor weights showed the same trend as tumor growth rates (Figure 1G). Tumor weights in OVA mRNA/CpG group were significantly smaller than that in OVA mRNA/Alum and OVA mRNA-alone groups (Figure 1H). Tumor sizes were also smaller in OVA mRNA/CpG group (Figure 1I). To be noted, 3 mice in OVA mRNA/CpG group developed no palpable tumors at the time of euthanasia and were kept alive. Although tumors finally developed in these mice, tumor initiation time was significantly delayed in this group (Figure 1J). The above studies indicate incorporation of CpG 1018 could significantly enhance cellular immune responses and anti-tumor immunity in preventive B16F10-OVA models.

### CpG 1018 increases OVA mRNA-induced anti-tumor immunity in therapeutic B16F10-OVA melanoma models

**Figure 2:**
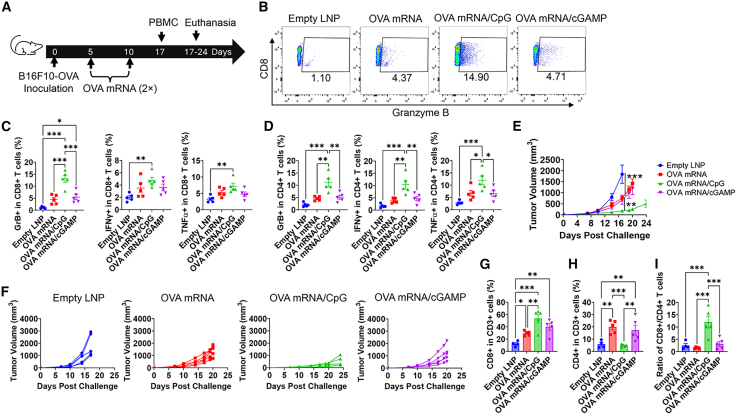
CpG 1018 enhances OVA mRNA-induced anti-tumor immunity in therapeutic B16F10-OVA models (A) Schematic illustration of experimental design in therapeutic B16F10-OVA melanoma models. (B–D) Cellular immune responses were analyzed 1 week after boosting. PBMCs were stimulated with OVA overnight followed by intracellular cytokine staining and flow cytometry analysis. Cells were first gated based on SSC and FSC and then CD4 and CD8 (Figure S2A). CD4^+^ and CD8^+^ T cells were then gated based on GrB, IFNγ, and TNFα (Figure S2A). Representative dot plots with gated GrB^+^ cells in CD8^+^ T cells were shown in (B). Percentages of GrB^+^ (left), IFNγ^+^ (middle), and TNFα^+^ cells (right) in CD8^+^ T cells were shown in (C). Percentages of GrB^+^ (left), IFNγ^+^ (middle), and TNFα^+^ cells (right) in CD4^+^ T cells were shown in (D). (E) Overall tumor growth rate in different groups. (F) Tumor growth rate of individual mice in each group. (G) Percentages of tumor-infiltrating CD8^+^ T cells in CD3^+^ T cells. (H) Percentages of tumor-infiltrating CD4+ T cells in CD3+ T cells. Gating strategies (G and H) were shown in Figure S2B. (I) Ratios of tumor-infiltrating CD8^+^ to CD4^+^ T cells. One-way ANOVA with Fisher’s LSD test was used to compare differences between groups in (C and D), and (G–I). Two-way ANOVA with Dunnett’s multiple comparisons test was used to compare differences between groups in (E). *n* = 5; ∗*p* < 0.05; ∗∗*p* < 0.01; ∗∗∗*p* < 0.001. Experiments were repeated twice with similar results.

The ability of CpG 1018 to enhance OVA mRNA-induced anti-tumor immunity was then explored in therapeutic B16F10-OVA models. Here, we compared CpG 1018 with cGAMP, a natural STING agonist capable of inducing type 1 IFN responses, to enhance OVA mRNA-induced anti-tumor immunity. Mice received prime/boost immunizations on days 5 and 10 after tumor inoculation as shown in Figure 2A. Cellular immune responses were analyzed 1 week after boosting. OVA mRNA/CpG induced vigorous expansion of GrB^+^CD8^+^ T cells in peripheral blood mononuclear cells (PBMCs) (Figure 2B), while OVA mRNA/cGAMP did not. Statistical analysis found OVA mRNA/CpG significantly increased percentages of GrB^+^ cells in CD8^+^ T cells as compared to other groups (left, Figure 2C). OVA mRNA/CpG also significantly increased percentages of IFNγ^+^ and TNFα^+^ cells in CD8^+^ T cells as compared to empty LNP (middle and right, Figure 2C). Similar phenomena were found in CD4^+^ T cells. OVA mRNA/CpG significantly increased percentages of GrB^+^, IFNγ^+^, and TNFα^+^ cells in CD4^+^ T cells as compared to other groups (Figure 2D). OVA mRNA significantly delayed B16F10-OVA tumor growth, while incorporation of CpG 1018 but not cGAMP more significantly delayed tumor growth (Figures 2E and 2F). Tumor-infiltrating lymphocytes (TILs) were then analyzed. As shown in Figure 2G, OVA mRNA/CpG induced the highest levels of tumor-infiltrating CD8^+^ T cells followed by OVA mRNA/cGAMP and then OVA mRNA. CD4^+^ T cell filtration showed a different trend. Incorporation of CpG reduced rather than increased frequencies of tumor-infiltrated CD4^+^ T cells when compared to OVA mRNA alone (Figure 2H). As such, incorporation of CpG profoundly increased the ratios of tumor infiltrating CD8^+^ to CD4^+^ T cells (Figure 2I). These results indicated CpG 1018 was also effective to enhance OVA mRNA-induced cellular immune responses and anti-tumor immunity in therapeutic B16F10-OVA models. As observed in preventive models, OVA mRNA/CpG 1018-induced transient weight loss (<6%, Figure S5B) in therapeutic B16F10-OVA models.

### CD8+ T cells contribute to the potency of CpG-adjuvanted OVA mRNA vaccine

**Figure 3:**
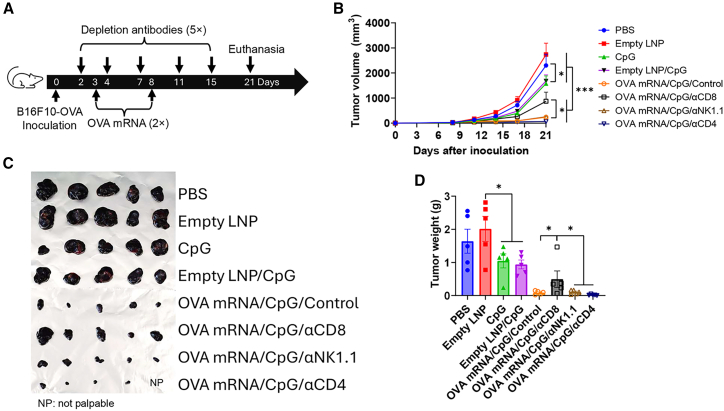
Crucial roles of CD8+ T cells in CpG 1018-adjuvanted OVA mRNA vaccine in therapeutic B16F10-OVA models (A) Schematic illustration of experimental design. (B) Tumor growth was monitored and compared among groups. (C) Tumors were harvested at the endpoint and imaged. (D) Tumor weight was compared among groups. *n* = 5. One-way ANOVA with Fisher’s LSD test was used to compare differences between groups in (D). Two-way ANOVA with Dunnett’s multiple comparisons test was used to compare differences between groups in (B). ∗*p* < 0.05; ∗∗*p* < 0.01; ∗∗∗*p* < 0.001. Experiments were repeated once with similar results.

Although anti-tumor immunity is traditionally mediated by CD8^+^ T cells, recent studies found CD4^+^ T cells can also play a role by reshaping tumor microenvironment.^24^ Previous studies found CpG ODN might also activate NK cells to suppress tumor growth.^25^ Next, we conducted cell depletion studies to explore potential roles of CD8^+^ T cells, CD4^+^ T cells, and NK cells in therapeutic efficacy of CpG 1018-adjuvanted OVA mRNA vaccine (Figure 3A). We also compared the relative potency of several control treatments (phosphate-buffered saline [PBS], CpG 1018, empty LNP, and empty LNP/CpG 1018) in the same study. Depletion antibodies were given 1 day before immunization and repeated for total 5 times during the course of the study. Near complete depletion of respective cells was confirmed by flow cytometry 1 week after the initiation of antibody-based cell depletion (Figure S6) and then repeated 1 week after with similar results (data not shown). OVA mRNA/CpG with isotype antibody injection significantly reduced tumor growth compared with PBS group (Figure 3B). CD8^+^ T cell depletion significantly increased tumor growth rate, while NK cell depletion showed no significant impact and CD4^+^ T cell depletion further reduced tumor growth rate despite a lack of statistically significant difference compared with isotype antibody injection (Figure 3B). These results indicated that CD8^+^ T cells played a crucial role in the observed therapeutic efficacy of CpG 1018-adjuvanted OVA mRNA vaccine, while NK and CD4^+^ T cells showed a minimal effect. CpG 1018 monotherapy led to a slight but statistically significant reduction of tumor growth, while empty LNP showed a minimal effect (Figure 3B). Empty LNP/CpG showed a similar effect to CpG 1018 monotherapy (Figure 3B). Tumor size (Figure 3C) and tumor weight (Figure 3D) at the endpoint showed the same trends as tumor growth rates. To be noted, one mouse showed no palpable tumor growth in CD4^+^ T cell-depleted group on day 21 (the endpoint) and was kept alive. This mouse developed measurable tumor 8 days later. Mice in CpG 1018-containing groups lost an average of less than 5% body weight (Figure S5C). Cell depletion antibodies against CD8, CD4, and NK1.1 delayed mouse body weight recovery by about 2 days after the second immunization (Figure S5C).

### CpG 1018 increases efficacy of NeoAg mRNA vaccine in therapeutic B16F10 melanoma models

**Figure 4:**
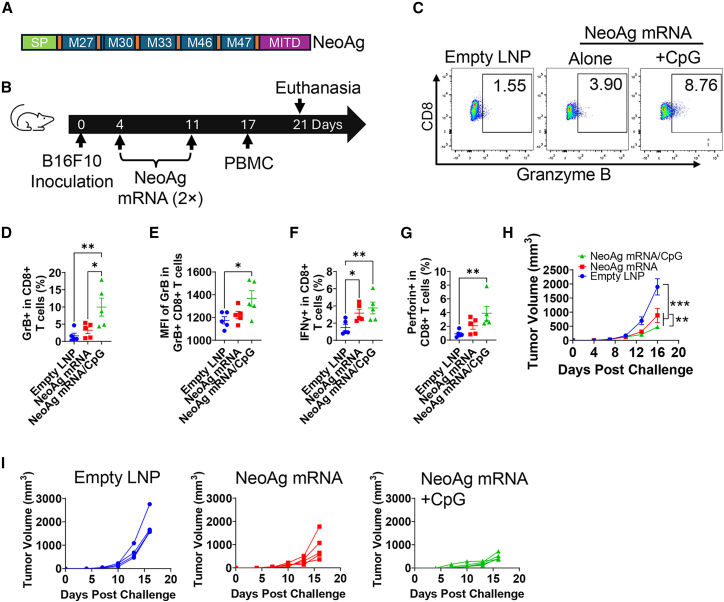
CpG 1018 increases NeoAg mRNA-induced anti-tumor immunity in therapeutic B16F10 model (A) Illustration of NeoAg mRNA design. (B) Illustration of experimental design. (C–G) PBMCs were collected 6 days after boost and stimulated with M27 and M30 peptide mixture overnight. Cells were collected 5 h later after addition of Brefeldin A and subjected to immunostaining and flow cytometry analysis. Cells were first gated based on FSC and SSC and then CD4 and CD8 expression. Cells were then gated based on GrB expression. Representative dot plots showing GrB^+^ cells in CD8^+^ T cells were shown in (C). Percentages of GrB^+^ cells in CD8^+^ T cells were shown in (D). MFI of GrB in GrB^+^CD8^+^ T cells was shown in (E). Percentages of IFNγ^+^ and perforin^+^ cells in CD8^+^ T cells were shown in (F) and (G), respectively. Gating strategies (C–G) were shown in Figure S2C. (H) Summary of tumor growth rate. (I) Tumor growth rate of individual mice in each group. One-way ANOVA with Fisher’s LSD test was used to compare differences between groups in (D–G). Two-way ANOVA with Fisher’s LSD test was used to compare differences between groups in (H). *n* = 5. ∗*p* < 0.05; ∗∗*p* < 0.01; ∗∗∗*p* < 0.001. Experiments were repeated once with similar results.

We further explored whether CpG 1018 was effective to enhance therapeutic efficacy of NeoAg mRNA vaccines, which were more relevant to that used in clinical trials.^1^^,^^3^^,^^4^^,^^26^ To explore this, well-recognized MHC I (M27 and M33) and MHC II NeoAg (M30, M46, and M47) of murine B16F10 melanoma (Table S1), linker sequences, signal peptide (SP), and MHC class I trafficking signal (MITD) (Table S2) were assembled to develop NeoAg mRNA vaccine for use in this study (Figure 4A).^24^^,^^27^ Corresponding DNA sequences were optimized for murine expression. NeoAg mRNA was similarly encapsulated in LNPs and found to have an average size of 99.4 nm (PDI: 0.08).

Mice were subcutaneously (s.c.) injected with B16F10 melanoma and intramuscularly (i.m.) immunized with NeoAg mRNA alone or admixed with CpG 1018 (NeoAg mRNA/CpG), or empty LNP. Immunization was repeated 1 week later (Figure 4B). Cellular immune responses were evaluated 6 days after boost. As shown in Figures 4C and 4D, GrB^+^ cell levels were significantly higher in NeoAg mRNA/CpG group than that in NeoAg mRNA-alone group. MFI of GrB was significantly increased in NeoAg mRNA/CpG but not NeoAg mRNA alone group (Figure 4E). Percentages of IFNγ^+^ cells were significantly increased in NeoAg mRNA alone or NeoAg mRNA/CpG group, while percentages of perforin^+^CD8^+^ T cells were only significantly increased in NeoAg mRNA/CpG group (Figures 4F and 4G). NeoAg mRNA significantly retarded tumor growth, while incorporation of CpG 1018 more significantly retarded tumor growth (Figures 4H and 4I). Similar to earlier findings, CpG 1018-adjuvanted NeoAg mRNA vaccination induced transient body weight loss (<3%, Figure S5D), supporting a good safety of CpG 1018-adjuvanted NeoAg mRNA vaccine.

### Impact of adjuvants on EGFP mRNA expression in cultured muscle cells and DCs

To understand how CpG 1018 augmented mRNA vaccine-induced CD8+ T cell responses, we first explored its impact on reporter EGFP mRNA translation in cultured muscle cells. Pseudouridine-modified EGFP mRNA was encapsulated in LNP with 93% efficiency. Human skeletal muscle cells were incubated with medium only, EGFP mRNA alone (encapsulated in LNP) or admixed with CpG 1018, cGAMP, AddaVax, or Alum at low and high concentrations recommended by manufacturers or typically used in the field.^28^^,^^29^^,^^30^

**Figure 5:**
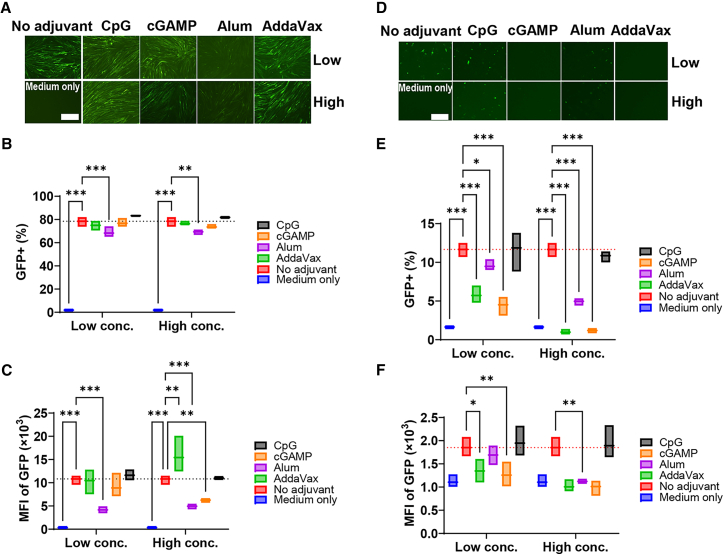
Impact of adjuvants on EGFP mRNA expression in muscle cells and BMDCs (A–C) Human skeletal muscle cells were incubated with EGFP mRNA alone or admixed with the respective adjuvants at low- or high-concentrations (conc.). Fluorescence images were taken 24 h later and shown in (A). Skeletal muscle cells were then harvested and subjected to flow cytometry analysis of percentages of GFP+ cells (B) and MFI of GFP (C). Gating strategies (B and C) were shown in Figure S2D. (D–F) Murine BMDCs were incubated with EGFP mRNA alone or mixed with the various adjuvants at low or high concentrations (conc.). Fluorescence images were taken 24 h later and shown in (D). BMDCs were then harvested and subjected to flow cytometry analysis of percentages of GFP^+^ cells (E) and MFI of GFP (F). Gating strategies (E and F) were shown in Figure S2E. Scale bars: 400 μm in (A) and 200 μm in (D). Two-way ANOVA with Fisher’s LSD test was used to compare differences of adjuvant-treated groups with no adjuvant control in (B) and (C) and (E–F). *n* = 3. ∗*p* < 0.05; ∗∗*p* < 0.01; ∗∗∗*p* < 0.001. Experiments were repeated once with similar results.

As shown in Figure 5A, fluorescence imaging found Alum at both concentrations and cGAMP at the high concentration suppressed EGFP expression. Flow cytometry analysis confirmed the above findings. Alum adjuvant at both concentrations significantly reduced GFP^+^ cell percentages (Figure 5B) and MFI of GFP (Figure 5C) when compared to no adjuvant control. cGAMP at the high concentration also significantly reduced MFI of GFP when compared to no adjuvant control (Figure 5C). CpG 1018 showed no significant impact on EGFP mRNA translation in muscle cells, while AddaVax at the high concentration significantly increased MFI of GFP (Figures 5B and 5C).

DCs are professional antigen-presenting cells with crucial roles to bridge innate and adaptive immunity.^31^ Antigenic mRNA may directly enter DCs and be translated into proteins to initiate antigen-specific immune responses. Next, we explored potential impacts of adjuvants on EGFP expression in murine bone marrow-derived dendritic cells (BMDCs). As shown in Figure 5D, the majority of adjuvants suppressed EGFP expression in BMDCs except CpG 1018, which showed a minimal effect. Flow cytometry analysis confirmed the above findings. cGAMP, Alum, and AddaVax, regardless of at high- or low-concentrations, significantly reduced percentages of GFP^+^ BMDCs (Figure 5E). AddaVax and cGAMP at low concentrations and Alum at the high concentration significantly reduced MFI of GFP in BMDCs (Figure 5F). Interestingly, CpG 1018 showed no significant impact on EGFP expression in BMDCs. These results indicate that CpG 1018 acted through other mechanisms to enhance mRNA vaccine-induced anti-tumor immunity.

### CpG 1018 increases DC maturation in the presence of OVA mRNA

**Figure 6:**
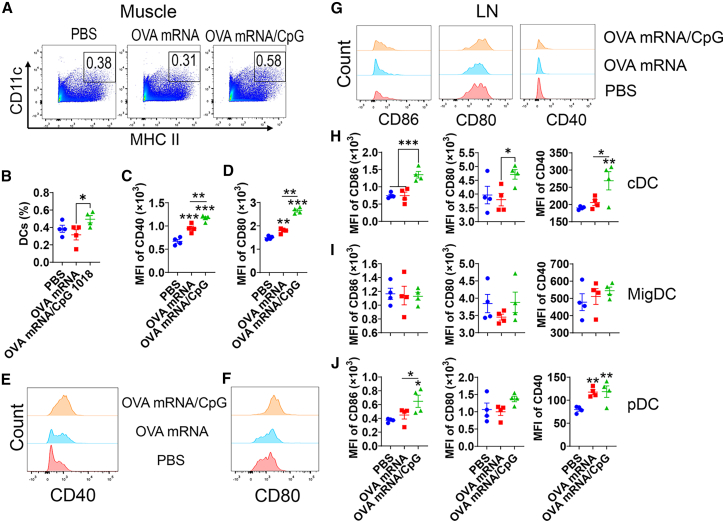
CpG 1018 enhances DC maturation in muscle and draining LNs in the presence of OVA mRNA Mice were subjected to IM injection of PBS, OVA mRNA, or OVA mRNA/CpG 1018. Muscle and draining inguinal LNs were collected 24 h later. Single-cell suspensions were prepared followed by immunostaining and flow cytometry analysis. Cells were first gated based on FSC and SSC and then CD11c and MHC II expression (Figure S2F). (A) Representative dot plots showing percentages of muscle DCs (CD11c^+^ MHC II^+^). (B) Comparison of muscle DC levels. (C) MFI of CD40 on muscle DCs. (D) MFI of CD80 on muscle DCs. (E) Representative histograms of CD40 levels on muscle DCs. (F) Representative histograms of CD80 levels on muscle DCs. (G) cDC, migDC, and pDC were identified based on relative CD11c and MHC II expression in draining LNs (Figure S2G). Representative histograms of CD86, CD80, and CD40 levels on cDC were shown. (H) MFI of CD86, CD80, and CD40 in cDC. (I) MFI of CD86, CD80, and CD40 in migDC. (J) MFI of CD86, CD80, and CD40 in pDC. One-way ANOVA with Fisher’s LSD test was used to compare differences between groups. *n* = 4; ∗*p* < 0.05; ∗∗*p* < 0.01; ∗∗∗*p* < 0.001. Experiments were repeated once with similar results.

Next, we explored DC maturation following intramuscular (IM) delivery of OVA mRNA or OVA mRNA/CpG. DC maturation is associated with increased expression of surface co-stimulatory molecules (CD40, CD80, and CD86), which engage CD28 and CD40L on T cells to promote T cell activation and differentiation.^32^ As shown in Figures 6A and 6B, OVA mRNA/CpG significantly increased muscle DC levels when compared to OVA mRNA alone. We also found OVA mRNA alone increased CD40 and CD80 levels on muscle DCs, while incorporation of CpG 1018 more significantly increased CD40 and CD80 levels on muscle DCs (Figures 6C–6F). Interestingly, OVA mRNA alone significantly increased CD86 expression, while incorporation of CpG 1018 reduced its expression to baseline levels (Figure S7A). These results indicated the ability of CpG 1018 to differentially regulate surface expression of co-stimulatory molecules on muscle DCs.

In draining inguinal lymph nodes (LNs), OVA mRNA alone or OVA mRNA/CpG did not significantly modify conventional DC (cDC), migratory DC (migDC), or pDC levels (Figure S7B). OVA mRNA/CpG but not OVA mRNA alone significantly increased CD40, CD80, and CD86 levels on cDC (Figures 6G and 6H). Similar CD40, CD80, or CD86 levels were found on migDC among groups (Figure 6I). OVA mRNA/CpG significantly increased CD40 and CD86 levels on pDC, while OVA mRNA alone only increased CD40 levels on pDC (Figure 6J). Similar results were obtained in draining popliteal LNs (data not shown).

### CpG 1018 increases OVA mRNA-induced cytokine release

**Figure 7:**
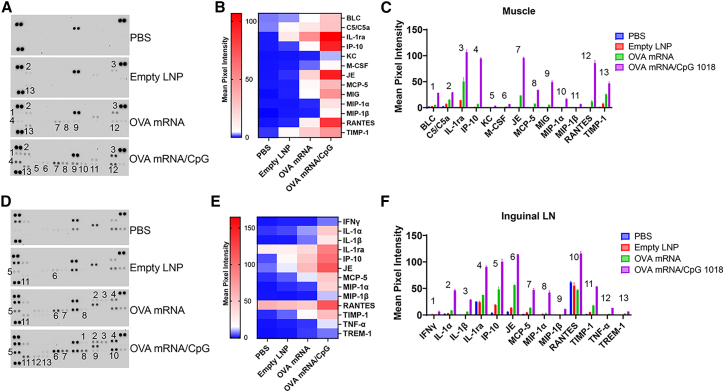
CpG 1018 increases OVA mRNA-induced cytokine release Mice (*n* = 4) were intramuscularly (i.m.) injected with PBS, empty LNP, OVA mRNA, or OVA mRNA/CpG 1018 mixture. Muscle and draining LNs were collected 24 h later and homogenized in T-PER buffer. Samples were pooled for measurement by cytokine array kits. (A) Membrane immunoblotting results of muscle samples. Dots with increased cytokine levels in OVA mRNA/CpG group as compared to PBS group were sequentially labeled with numbers. Dots with increased cytokine levels in empty LNP and OVA mRNA groups were then labeled with corresponding numbers from OVA mRNA/CpG group. (B) Heatmap analysis of cytokine expression in muscle samples. (C) Mean pixel intensities of cytokines in muscle samples. (D) Membrane immunoblotting results of draining LN samples. Figures were similarly labeled with numbers as in (A). (E) Heatmap analysis of cytokine expression in draining LN samples. (F) Mean pixel intensities of cytokine expression in draining LN samples. Mean pixel intensities of cytokine spots in (A) were obtained with ImageJ and the average values were used to prepare the heat maps in (B) and (E). Error bars in (C) and (F) reflected the differences of cytokine levels of duplicate spots in (A) and (D). Number labels in (A) and (D) matched that listed in (C) and (F), respectively.

Adjuvants may also induce local cytokine/chemokine release to augment innate or adaptive immune responses.^23^ Cytokine release may provide signal 3 to induce more potent T cell responses besides necessary signals 1 (antigen) and 2 (co-stimulatory molecules),^33^ while chemokine release may recruit innate immune cells to the site of inflammation.^34^ To explore this, we evaluated local cytokine/chemokine release following IM delivery of OVA mRNA alone or OVA mRNA/CpG. As shown in Figures 7A–7C, empty LNP induced weak expression of IL-1ra, C5/C5a, and TIMP-1 in the muscle. OVA mRNA increased the expression of these cytokines and also induced unique expression of JE, RANTES, IP-10, MCP-5, MIG, and BLC. JE (or MCP-1) and MCP-5 belong to CC chemokine family and attract monocytes by binding to CCR2 receptor.^35^^,^^36^ RANTES (or CCL5) is a proinflammatory chemokine and mediate leukocyte migration by binding to various CCR receptors including CCR5.^37^ MIG (or CXCL9) and IP-10 (or CXCL10) belong to CXC chemokine family and are IFNγ-induced with important roles to recruit activated T cells to the site of inflammation.^38^ Incorporation of CpG 1018 significantly increased the expression of these chemokines and also induced unique expression of MIP-1α, MIP-1β, M-CSF (macrophage colony-stimulating factor), and KC. MIP-1α (or CCL3) and MIP-1β (or CCL4) belong to CC chemokine family and are secreted by macrophages with important roles to recruit monocytes, T cells, and NK cells by binding to CCR5 receptor.^39^ M-CSF plays crucial roles in proliferation, differentiation, and survival of monocytes and macrophages.^40^ High levels of JE/MCP-5 and M-CSF after incorporation of CpG 1018 were in good agreement with the increased DC levels in the muscle (Figure 6B), presumably due to the recruitment and differentiation of monocytes into DCs.

The same phenomena were observed in draining LNs (Figures 7D–7F). Empty LNP induced weak expressions of IP-10, JE, MCP-5, and TIMP-1. OVA mRNA increased the expression of these cytokines and also induced unique expression of IL-1α, IL-1β, and IL-1ra. This finding was in line with a prior report that IL-1 and IL-1ra are key regulators of the inflammatory responses to RNA vaccines.^41^ Strong induction of IL-1ra antagonizes the proinflammatory effects of IL-1α and IL-1β and protects mice from cytokine-mediated cytotoxicity.^41^ Incorporation of CpG 1018 significantly increased expression of these cytokines and also induced unique expression of MIP-1α, RANTES, MIP-1β, TNF-α, triggering receptor expressed on myeloid cells (TREM)-1, and IFNγ, among which TNF-α, TREM-1, and IFNγ are new cytokines uniquely induced in the draining LNs. TNF-α and IFNγ are known downstream cytokines of CpG-mediated TLR9 activation.^42^ TREM-1 is the first TREM identified and has a role to amplify monocyte and granulocyte responses to microbial products.^43^ There were 8 cytokines/chemokines induced by OVA mRNA/CpG in both muscle and draining LNs: IL-1ra, IP-10, JE, MCP-5, MIP-1α, MIP-1β, RANTES, and TIMP-1. The above results indicated OVA mRNA alone could induce local inflammation (e.g., cytokine/chemokine release), while incorporation of CpG 1018 induced more vigorous local inflammation.

### OVA mRNA/CpG increases functionality of CD8+ T cells

**Figure 8:**
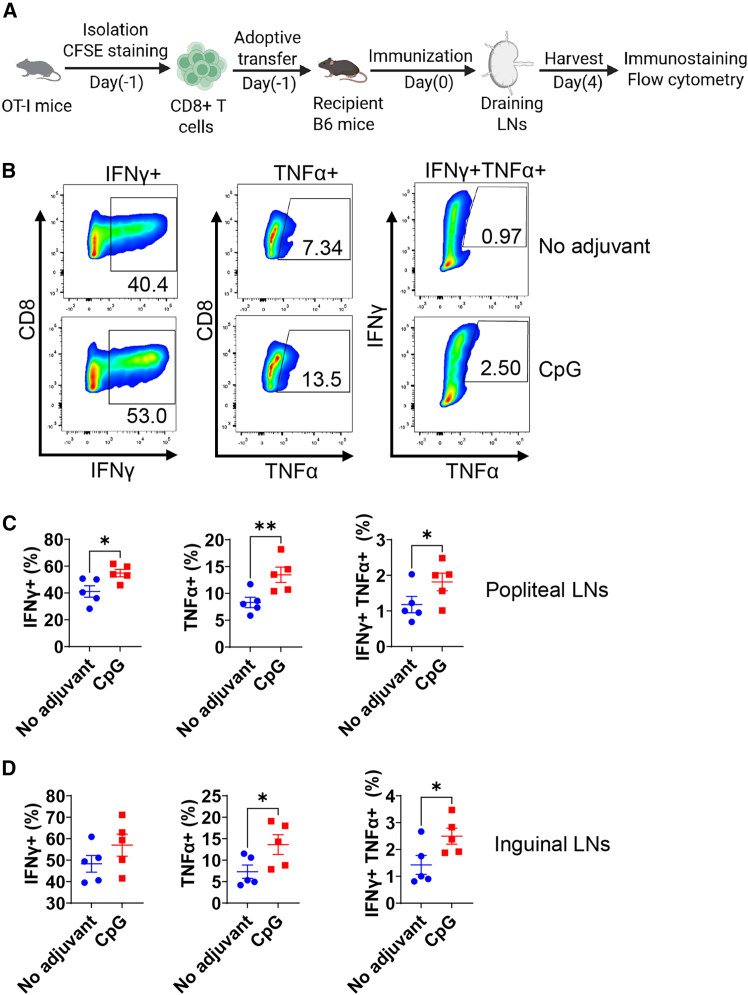
CpG 1018 increases cytokine-secreting OT-I T cells following adoptive transfer OT-I T cells were magnetically purified from OT-I mice, stained with CFSE, and adoptively transferred to naive C57BL/6 mice. After 24 h, recipient mice were i.m. immunized with OVA mRNA alone or OVA mRNA/CpG 1018. After 4 days, draining popliteal and inguinal LNs were collected and stimulated overnight with OVA followed by immunostaining and flow cytometry analysis. Cells were first gated based on FSC and SSC and then CD4 and CD8. CD8+ T cells were then gated based on CFSE. CFSE+ cells were further analyzed for IFNγ and TNFα expression. Gating strategies were shown in Figure S2H. (A) Illustration of experimental procedures. (B) Representative dot plots showing IFNγ and TNFα single-positive or double-positive cells. (C) Percentages of IFNγ and TNFα single-positive or double-positive cells in CFSE+ cells in popliteal LNs. (D) Percentages of IFNγ and TNFα single-positive or double-positive cells in CFSE+ cells in inguinal LNs. One-tailed Student’s *t* test was used to compare differences between groups in (B) and (C). *n* = 5; ∗*p* < 0.05; ∗∗*p* < 0.01. Experiments were repeated once with similar results.

Significantly increased signals 2 and 3 in DCs were expected to induce more potent CD8^+^ T cell responses in draining LNs. To explore this, carboxyfluorescein diacetate succinimidyl ester (CFSE)-stained OT-I T cells (recognizing H-2K^b^-restricted OVA_257-264_ peptide) were adoptively transferred to C57BL/6 mice followed by IM OVA mRNA immunization with or without CpG 1018 (Figure 8A). As shown in Figures 8B and 8C, incorporation of CpG 1018 significantly increased percentages of IFNγ^+^, TNFα^+^, and IFNγ^+^TNFα^+^ OT-I T cells in popliteal draining LNs. Similar trends were observed in downstream inguinal draining LNs (Figure 8D). Incorporation of CpG 1018 significantly increased TNFα^+^ and IFNγ^+^TNFα^+^ OT-I T cells in inguinal draining LNs (Figure 8D). These results indicated incorporation of CpG 1018 in OVA mRNA vaccination could significantly increase functionality of antigen-specific CD8^+^ T cells in the draining LNs.

Overall, we propose a model to explain CpG 1018-enhanced mRNA vaccine efficacy in anti-tumor immunotherapy as shown in Figure S8. CpG 1018 significantly enhances DC maturation and cytokine secretion in muscle and draining LNs, contributing to augmented CD8+ T cell expansion and secretion of GrB, perforin, IFNγ, and TNFα, leading to enhanced anti-tumor immunity. Our studies also found CD4+ T cells and NK cells played no significant role in CpG 1018-augmented mRNA vaccine efficacy in anti-tumor therapy.

## Discussion

Strategies are under sought to further enhance personalized cancer mRNA vaccine efficacy and induce long-term remission. This study found CpG 1018 adjuvant approved for use in HEPLISAV-B could vigorously enhance mRNA vaccine-induced anti-tumor immunity in preventive and therapeutic melanoma models. OVA mRNA vaccination in the presence of CpG 1018 significantly delayed B16F10-OVA tumor initiation and growth in preventive models. CpG 1018 also augmented OVA mRNA or NeoAg mRNA vaccine efficacy in therapeutic B16F10 melanoma models. OVA mRNA alone suppressed B16F10-OVA melanoma growth by 59%, while OVA mRNA/CpG suppressed tumor growth by 90% (Figure 2E). NeoAg mRNA alone suppressed B16F10 melanoma growth by 53%, while NeoAg mRNA/CpG 1018 suppressed tumor growth by 75% (Figure 4H). These promising results support incorporation of CpG 1018 to enhance NeoAg mRNA vaccine efficacy in tumor therapy.

CpG 1018 was found to augment mRNA vaccine-induced cytokine/chemokine release, which could recruit peripheral innate immune cells to augment mRNA vaccine-induced adaptive immune responses and also provide additional signals to reshape and enhance T cell activation. The adjuvant effects of CpG 1018 can also be ascribed to its ability to induce DC maturation. DC maturation increases surface expression of co-stimulatory molecules to engage respective receptors on T cells and enhance T cell activation and differentiation. Our data suggested CpG 1018 mainly acted on myeloid DC or cDC but not pDC to achieve its adjuvant effects. In support, pDC depletion during vaccination played a non-essential role in CpG 1018-augmented NeoAg mRNA vaccination in preventive B16F10 melanoma models (data not shown). Consistent with the induction of potent innate immunity, mRNA vaccine in the presence of CpG 1018 significantly increased GrB, IFNγ, perforin, and TNFα-secreting CD8+ T cells in -*in vivo* animal models. Incorporation of CpG 1018 in OVA mRNA vaccination was found to significantly increase tumor-infiltrating CD8^+^ T cells but significantly reduce tumor-infiltrating CD4^+^ T cells, leading to a significant increase of tumor-infiltrating CD8^+^ to CD4^+^ T cell ratio (∼8-folds). These results were in line with cell depletion studies. We found depletion of CD8^+^ T cells significantly reduced therapeutic efficacy of CpG 1018-adjuvanted OVA mRNA, while depletion of CD4^+^ T cells increased therapeutic efficacy of CpG 1018-adjuvanted OVA mRNA though to a non-statistically significant level. These findings were in line with prior reports that high ratios of tumor-infiltrating CD8^+^ to CD4^+^ T cells were often associated with higher response rates to treatment and better therapeutic outcomes in melanoma,^44^ ovarian cancer,^45^ and breast cancer.^46^ The unfavorable effects of tumor-infiltrating CD4^+^ T cells might be due to regulatory T cells (Tregs).^45^ Our studies found CpG 1018 monotherapy led to a slight but statistically significant reduction of tumor growth. Yet NK cells were less likely to be involved considering depletion of NK cells showed no impact on B16F10-OVA tumor growth in OVA mRNA/CpG group. This is likely due to the use of type B CpG with relatively weak ability to stimulate IL-12 and type 1 IFN responses required for NK cell activation.^47^ The anti-tumor efficacy of CpG 1018 when delivered alone is likely due to its ability to modulate tumor microenvironment. Our unpublished data found intratumoral injection of CpG 1018 could significantly increase tumor-infiltrating CD8 to CD4+ T cell ratios.

Our studies found cGAMP was not effective to enhance OVA mRNA-induced cellular immune responses and anti-tumor immunity in therapeutic B16F10-OVA tumor models. This might reflect the limited cytosolic delivery of free cGAMP to access STING. In contrast, mRNA delivered via heterocyclic lipids capable of stimulating STING or co-delivered with cGAMP in a nanovaccine elicited more potent anti-tumor immunity.^48^^,^^49^ These studies indicated importance of nano-formulation or alternative STING pathway activation in boosting mRNA vaccine-induced anti-tumor immunity. Our studies found AddaVax was less potent than CpG 1018, while Alum was completely ineffective to enhance OVA mRNA-induced anti-tumor immunity in preventive B16F10-OVA melanoma models. These results were in line with their major roles to enhance vaccine-induced antibody responses with limited ability to potentiate cellular immune responses. Yet, we found all these adjuvants including cGAMP were not effective to enhance mRNA vaccine-induced antibody responses. The inability of these adjuvants to enhance OVA mRNA-induced antibody responses reflected the unique nature of mRNA vaccines that lack protein-based vaccine antigens at the time of vaccine delivery.

CpG 1018-adjuvanted mRNA vaccines induced a temporary weight loss of less than 6% in our studies. Our unpublished protein immunization in the presence of CpG 1018 adjuvant also induced a similar level of temporary weight loss. These results indicated weight loss was mainly due to CpG 1018 adjuvant. Considering CpG 1018 has been used in HEPLISAV-B in humans with good safety profiles, we are not expecting significant safety concerns to use CpG 1018 to boost mRNA vaccine-induced anti-tumor immunity.

Our data indicate the promise of CpG 1018 adjuvant to enhance NeoAg mRNA vaccine-induced anti-tumor immunity. It is also highly rational to combine CpG 1018-adjuvanted NeoAg mRNA vaccine with ICIs to achieve better therapeutic efficacy due to the ability of ICIs to increase cytotoxicity of tumor-specific T cells.^50^ Although we only tested adjuvant potency of CpG 1018 in murine melanoma models, CpG 1018 is promising to show similar adjuvant effects to boost NeoAg mRNA vaccine efficacy against other tumor types considering adjuvant effects of CpG 1018 are less likely to be affected by NeoAg sequences. Overall, our data support the effectiveness of a clinically used CpG 1018 adjuvant to augment mRNA vaccine-induced anti-tumor immunity in murine melanoma models and warrants further investigation.

## Materials and methods

### Reagents

CleanCap OVA and EGFP mRNA (5 moU) were purchased from TriLink Biotechnologies (San Diego, CA, USA). CleanCap B16F10 NeoAg mRNA (5 moU) and CpG 1018 were custom synthesized by TriLink Biotechnologies (San Diego, CA, USA). cGAMP (vac-nacga23), Alum (vac-alu-250), AddaVax (vac-adx-10), endotoxin-free OVA (vac-pova) were all purchased from InvivoGen (San Diego, CA, USA). CellTrace CFSE Cell proliferation Kit (C34554), Fixable Viability Dye eFluor 450 (65-0863-14), TMB substrate (34028), T-PER Reagent (78510), protease and phosphatase inhibitor (78442), RiboGreen RNA Quantification Kit (R32700) were purchased from Thermo Fisher Scientific, Waltham, MA, USA). Fluorescence-conjugated antibodies, anti-CD28 antibody, Brefeldin A were obtained from BioLegend (San Diego, CA, USA). Horseradish peroxidase (HRP)-conjugated sheep anti-mouse IgG secondary antibody was purchased from Cytiva (Cytiva Cat# NA931, RRID:AB_772210). HRP-conjugated goat anti-mouse IgG1 (Thermo Fisher Scientific Cat# PA1-74421, RRID:AB_10988195) and goat anti-mouse IgG2c (Bethyl Cat# A90–136 P, RRID:AB_67165) antibodies were purchased from Thermo Fisher Scientific (Waltham, MA, USA) and Bethyl Laboratories (Montgomery, TX, USA), respectively. Naive CD8a^+^ T cell Isolation Kit was purchased from Miltenyi Biotec (130-096-543). LNP-102 Exploration Kit (35425) was purchased from Cayman Chemical (Ann Arbor, MI, USA). Collagenase D was purchased from Sigma (11088866001) and Dispase II was purchased from Thermo Fisher Scientific (17105041, Waltham, MA, USA). B16F10-OVA cells were obtained as a kind gift from Dr. Jeffrey A Hubbell at University of Chicago. B16F10 cells were purchased from ATCC (CRL-6475, Gaithersburg, MD, USA). M27 and M30 NeoAg peptides were custom-synthesized by GenScript (Piscataway, NJ, USA). Mouse Cytokine Array Panel A was purchased from Bio-Techne (ARY006, Minneapolis, MN, USA). Depletion anti-mouse CD8 antibodies (BE0061), anti-mouse CD4 antibodies (BE0003), and anti-mouse NK1.1 antibodies (BE0036) were obtained from Bio X Cell (Lebanon, NH, USA).

### Mice

C57BL/6 mice (male, 6–8 weeks old) were purchased from the Jackson Laboratory (Bar Harbor, ME, USA). OT-I mice (003831) were purchased from the Jackson Laboratory (Bar Harbor, ME, USA) and self-bred for use in this study. Mice were housed in the animal facilities of University of Rhode Island. The mice were anesthetized by inhalational exposure of 2.5%–3% isoflurane for immunization. Mice were euthanized in their home cages by delivering compressed CO_2_ in gas cylinders via an Euthanex lid in the procedure room. All animal procedures were approved by the Institutional Animal Care and Use Committee of University of Rhode Island.

### Immunization

Mice were i.m. injected with 5 μg OVA mRNA or NeoAg mRNA encapsulated in LNP alone or admixed with CpG 1018 (40 μg), cGAMP (20 μg), AddaVax (1:1 vol:vol) or Alum (1:1 vol:vol) into the thigh muscles of the hind limb. Mice were i.m. injected with empty LNP or PBS to serve as control or otherwise specified. Immunizations were repeated similarly as in priming immunizations at indicated intervals. Mouse body weight was monitored daily for 1–2 weeks after immunizations as an indicator of systemic safety.

### LNP mRNA vaccine preparation and characterization

Lipids were dissolved in pure ethanol at molar ratios of 50:10:38.5:1.5 (SM-102:1,2-DSPC:cholesterol:DMG-PEG 2000). The lipid mixture was mixed with mRNA dissolved in 50 mM sodium acetate (pH 5.0) at a volume ratio of 3:1 (aqueous: ethanol) using the NanoAssemblr Ignite system (Precision Nanosystems) at a total flow rate of 9 mL/min, followed by dialysis against PBS and then 8.7% sucrose in PBS. The lipid to ODN (w/w) ratio was 10:1. Following dialysis, the mRNA LNPs were sterilized by passing through 0.22 μm filter and concentrated with 3 kDa MW-cutoff centrifugal filters. The sizes of mRNA LNPs were measured by dynamic light scattering (DLS) analysis with Malvern Zetasizer. The morphology of LNPs was characterized by cryogenic electron microscopy (cryo-EM). Empty LNPs were similarly prepared and characterized as mRNA-encapsulated ones except no mRNA was added to aqueous phase.

### LNP mRNA EE

LNPs were treated with 2% Triton X-100 to disrupt the lipid structure to measure total mRNA levels, while untreated LNPs were used to measure unencapsulated mRNA levels. mRNA levels were measured with RiboGreen RNA Quantification Kit. The (EE%) was calculated as

(Equation 1) $$\frac{total\;mRNA-unencapsulated\;mRNA}{total\;mRNA}\times 100\%.$$

### Human skeletal muscle culture

Primary human skeletal muscle cells (ATCC PCS-950-010) were first expanded in complete growth media prepared by adding one Primary Skeletal Cell Muscle Growth Kit (ATCC PCS-950-040) to one bottle of Mesenchymal Stem Cell Basal Medium (ATCC PCS-500-030). Primary human skeletal muscle cells suspended in complete growth medium were seeded into fibronectin-coated 96-well plates at the recommended density of 50,000 cells/cm^2^. To prepare fibronectin-coated 96-well plates, 100 μL fibronectin (Corning 354008) was added per well with a final concentration of 5 μg/cm^2^. Fibronectin solution was then removed. The plates were allowed to air dry for 45 min before use. After 24 h, complete growth medium was replaced with differentiation medium (ATCC PCS-950-050) and cells were cultured for 4 days before use.

### BMDCs

Murine BMDCs were prepared as shown in our previous reports.^51^^,^^52^ In brief, bone marrows were obtained from femur and tibia of C57BL/6 mice. Single-cell suspensions were obtained after vigorous pipetting. Red blood cells (RBCs) were removed by ACK lysis buffer. Cells were cultured in RPMI 1640 medium supplemented with recombinant mouse GM-CSF (415-ML-020/CF) and IL4 (404-ML-025/CF), both from R&D Systems. Half volumes of medium were replaced every other day. Cells were used on day 6.

### EGFP mRNA transfection of cultured cells

BMDCs were seeded at 10^6^/mL into 96-well plates (200 μL/well). BMDCs and skeletal muscle cells were treated with medium only, 0.4 μg/mL LNP (EGFP mRNA) alone or in the presence of below adjuvants at two concentrations: CpG (1 and 10 μg/mL), cGAMP (1 and 10 μg/mL), Alum (10 and 100 μg/mL), and AddaVax (0.05 and 0.5%). Fluorescence images were taken 24 h after incubation in the EVOS M7000 microscope. Skeletal muscles were then harvested and subjected to flow cytometry analysis without further staining.

### Mouse cytokine array

Cytokine levels in muscle and LNs were measured with the Mouse Cytokine Array Panel A (ARY006, R&D Systems) as previously described.^53^ In brief, muscle and LNs were collected and homogenized in T-PER buffer supplemented with protease and phosphatase inhibitor followed by centrifugation at 10,000× *g* for 5 min at 4°C. Supernatants were pooled for analysis of cytokine levels. Briefly, membranes coated in duplicate with capturing antibodies against 40 cytokines were blocked and then incubated with tissue homogenates in the presence of a cocktail of biotinylated detection antibodies. After washing, membranes were incubated with Streptavidin-HRP for 30 min and then Chemi Reagent Mix. Membranes were imaged using iBright Imaging System (CL750, Thermo Fisher Scientific). ImageJ was used to quantify the mean pixel intensity for each dot on the blot.

### Muscle single-cell suspension preparation

Muscle single-cell suspensions were prepared as previously described with slight modifications.^54^ Briefly, quadriceps muscles were collected 18 h after immunization, cut into small pieces, and digested in 5 mL muscle dissociation buffer (Hanks’ Balanced Salt Solution (HBSS) supplemented with 1% penicillin/streptomycin, 10% fetal bovine serum (FBS), and 725 U/mL collagenase type 2) at 37°C for 1 h with constant shaking (100 rpm). Digestion was stopped by adding 10 mL cold wash medium (HBSS supplemented with 1% penicillin/streptomycin and 10% FBS), followed by centrifugation at 525× g for 5 min at room temperature. After removing 11 mL supernatants, 0.5 mL collagenase type 2 (100 U/mL) and 0.5 mL Dispase (1.1 U/mL) were added to resuspend the pellets followed by further digestion at 37°C for 30 min with constant shaking (100 rpm). Samples were then passed through an 18-gauge needle 10–12 times to break any remaining tissue. Around 10 mL cold wash medium was added followed by centrifugation at 525× g for 5 min at room temperature. 11 mL supernatants were removed. The remaining 4 mL volume was gently mixed and passed through 70 μm cell strainer. Cells were washed with fluorescence-activated cell sorting (FACS) buffer (PBS supplemented with 2% FBS) followed by immunostaining.

### Adoptive transfer

Purification of OT-I cells, CFSE staining, and adoptive transfer were performed according to our previous report.^55^ Briefly, spleens from OT-I transgenic mice were harvested, followed by passing thorough 70 μm cell strainers to obtain single-cell suspensions. RBCs were lysed using ACK lysis buffer. Cells were then subjected to magnetic beads-based negative purification of naive CD8^+^ T cells with a commercial kit (130–096–543, Miltenyi Biotec). The purified naive CD8^+^ T cells (OT-I cells) were stained with 10 μM CFSE (C34554, Thermo Fisher Scientific) at 37°C for 20 min. CFSE-stained OT-I cells were thoroughly washed in PBS and then intravenously (i.v.) injected into C57BL/6 mice (10^6^ OT-I cells per mouse). Mice were immunized 24 h later. Draining LNs were collected 4 days after immunization for further analysis.

### PBMC preparation and stimulation

A small volume of blood (∼50 μL) was collected into heparinized tubes followed by RBC lysis using ACK lysis buffer (118-156-101, Quality Biological). For intracellular cytokine staining, PBMCs were stimulated with 10 μg/mL OVA or a mixture of M27 and M30 peptides (1 μg/mL each) in the presence of 4 μg/mL anti-CD28 antibodies (37.51) (BioLegend Cat# 102116, RRID:AB_11147170) overnight. Next day, Brefeldin A (420601, BioLegend) was added 5 h before cell harvest.

### Cell depletion

CD8+ T cells, CD4+ T cells, and NK cells were depleted by intraperitoneal (i.p.) injection of anti-mouse CD8, CD4, and NK1.1 antibodies (200 μg per mouse) at indicated times. Successful depletion of respective cells was confirmed by flow cytometry analysis of PBMCs 1 and 2 weeks after initiation of cell depletion.

### Tumor inoculation

B16F10-OVA or B16F10 cells were cultured in complete DMEM medium and harvested at around 80% confluency. Cells were washed in cold PBS for 3 times. Around 10^6^ B16F10-OVA cells in 100 μL were s.c. injected into the right flank of mice in preventive tumor models. About 2 × 10^5^ B16F10-OVA or B16F10 cells in 100 μL were s.c. injected into the right flank of mice in therapeutic tumor models.

### Tumor size measurement

Tumor size was measured with a digital caliper. Tumor volume was calculated according to the formula $Volume=\frac{1}{2}\times Length\times {Width}^{2}$ as previously described.^52^^,^^55^^,^^56^ The mice were euthanized after reaching humane endpoint or otherwise specified.

### TIL preparation

TILs were prepared following a published protocol with slight modifications.^57^ In brief, tumor tissues were harvested and minced into small pieces and digested with 0.2% Collagenase D (11088866001, Sigma-Aldrich) and 0.6 U Dispase (17105-041, Life Technologies) at 37°C with constant shaking at 100 rpm for 1 h. The digested tissues were passed through 70 μm cell strainers and resuspended with RPMI-1640 medium to obtain a total volume of 10 mL. Around 4 mL Ficoll-Paque medium was added to a 15-mL conical tube followed by gently adding 10 mL tumor cell suspensions on the top. The tubes were centrifuged at 1,025× g for 20 min at room temperature with acceleration and deceleration off. The mononuclear cell layers at the interface were collected and washed with RPMI 1640 medium followed by immunostaining.

### Immunostaining and flow cytometry

BMDCs, tissue muscle cells, and LN cells were stained with fluorescence-conjugated antibodies against CD11c (N418, PE), CD40 (3/23, pacific blue), CD80 (16-10A1, PerCP-Cy5.5), CD86 (GL-1, FITC), and MHC II (M5/114.15.2, AF647) to evaluate DC maturation status. Cells isolated from draining LNs in adoptive transfer experiments were stimulated with OVA and then stained with fluorescence-conjugated anti-CD4 (GK1.5, APC) and anti-CD8 antibodies (53-6.7, PerCP-Cy5.5). Cells were then fixed, permeabilized, and stained with fluorescence-conjugated anti-IFNγ (XMG1.2, BV421) and anti-TNFα antibodies (MP6-XT22, BV510). PBMCs from tumor vaccine studies were stimulated with OVA or NeoAg peptides and then stained with fluorescence-conjugated anti-CD4 (GK1.5, PerCP-Cy5.5) and anti-CD8 antibodies (53-6.7, APC). Cells were then fixed, permeabilized, and stained with fluorescence-conjugated anti-Granzyme B (NGZB, PE), anti-IFN-γ (XMG1.2, FITC), and anti-IL4 (11B11, BV421), or anti-TNFα (MP6-XT22, pacific blue), or anti-perforin antibodies (S16009A, BV421). TILs were stimulated with OVA and then stained with fixable viability dye eFluor 450, fluorescence-conjugated anti-CD3 (17A2, BV510), anti-CD4 (GK1.5, APC), and anti-CD8 antibodies (53-6.7, PerCP-Cy5.5). Cells were subjected to flow cytometry analysis in BD FACSVerse. Data were analyzed by FlowJo software.

### ELISA

Serum antibody titers were measured by enzyme-linked immunosorbent assay (ELISA) as previously described.^53^^,^^56^ In brief, serum samples were subject to 4-fold serial dilutions and added to 96-well plates pre-coated with 10 μg/mL OVA. After 90 min incubation and washing, HRP-conjugated anti-mouse IgG, IgG1, or IgG2c secondary antibodies were added. After 1 h incubation and washing, TMB substrates were added. The optical absorbance (OD_450nm_) was read in a microplate reader after the addition of 2 M H_2_SO_4_.

### Statistics analysis

Values were expressed as mean ± SEM (standard error of the mean). One-way analysis of variance (ANOVA) with Tukey’s multiple comparison or Fisher’s least significant difference (LSD) test was used to compare differences for more than two groups or otherwise specified. Two-way analysis of variance (ANOVA) with Tukey’s multiple comparison or Fisher’s LSD test was used to analyze data with two variables or otherwise specified. *p* value was calculated by PRISM software (GraphPad, San Diego, CA, USA) and considered significant if it was <0.05.

## Data and code availability

The raw/processed data required to reproduce these findings are available from the corresponding author upon request.

## Acknowledgments

This work was partially supported by the University of Rhode Island College of Pharmacy Pilot funding (to X.C.). Microplate reader and BD FACSVerse used in this work were supported by an 10.13039/501100024771Institutional Development Award (IDeA) from the 10.13039/100000057National Institute of General Medical Sciences of the 10.13039/100000002National Institutes of Health grant P20GM103430. Cyro-EM images were captured at RI Consortium for Nanoscience and Nanotechnology Lab supported by the 10.13039/100000001National Science Foundation (NSF) 10.13039/100005714EPSCoR cooperative agreement #OIA-1655221.

## Author contributions

X.C. designed experiments; Y.L., J.R.N., L.A., X.K., Y.S., and E.V. conducted experiments and acquired data; X.C. and Y.L. analyzed data; X.C. wrote the manuscript.

## Declaration of interests

The authors declare no competing interests.

## Supplemental information

Document S1. Figures S1–S8, Tables S1, and S2

Document S2. Article plus supplemental information
