# Supplementary material for: CpG 1018 augments mRNA vaccine-induced anti-tumor immunity by potentiating CD8+ T cell responses
Source: Mol Ther Oncol. 2026 May 9;34(2):201230. doi: 10.1016/j.omton.2026.201230 (PMC13233743; doi:10.1016/j.omton.2026.201230)
Supplement: Document S1. Figures S1–S8, Tables S1, and S2 [file mmc1.pdf]

OMTON, Volume *34*

Supplemental information

CpG 1018 augments mRNA vaccine-induced anti-tumor immunity by potentiating CD8+ T cell responses

Yibo Li, Jayachandra Reddy Nakkala, Labone Akter, Xinliang Kang, Yuna Song, Elise VanLuinen, and Xinyuan Chen

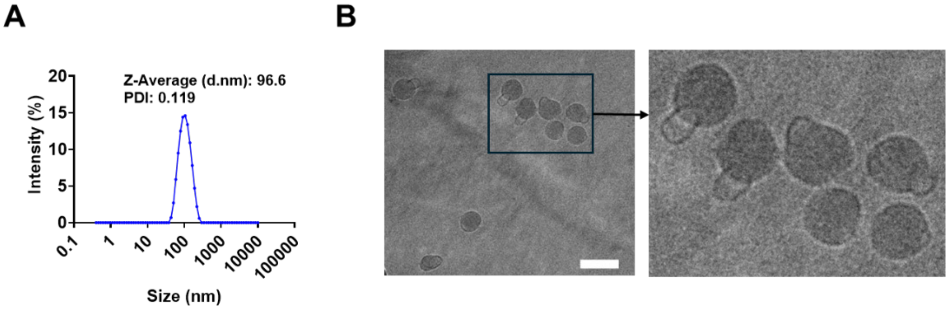

Figure S1. DLS (A) and Cryo-EM (B) analysis of LNP(OVA mRNA) (Scale: 200 μm in B)

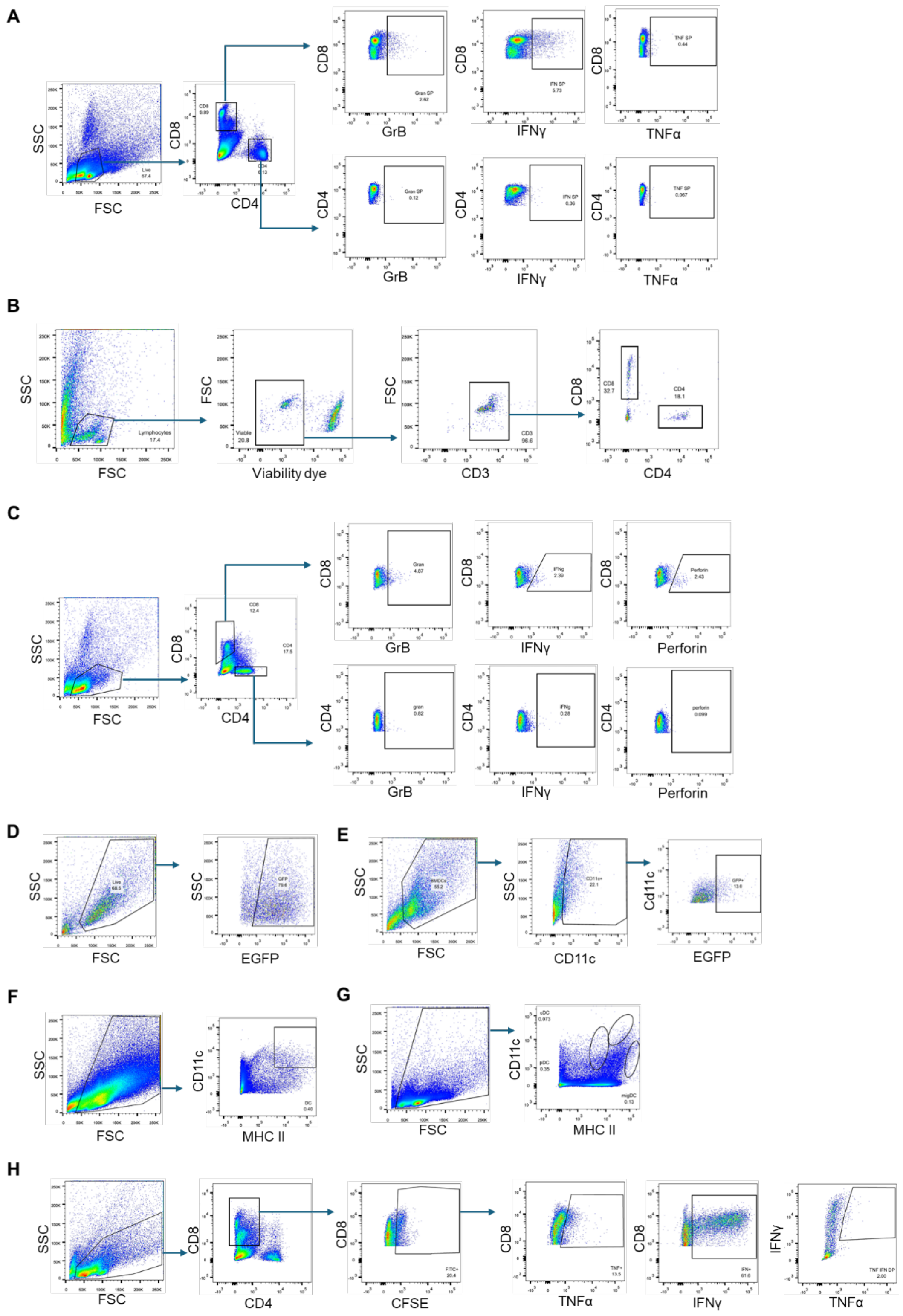

Figure S2. Gating strategies

A. Gating strategies for Fig.1B-E and 2B-D. B. Gating strategies for Fig.2G-H. C. Gating strategies for Fig.4C-G. D. Gating strategies for Fig.5B-C. E. Gating strategies for

Fig.5E-F. F. Gating strategies for Fig.6A. G. Gating strategies for Fig.6G. H. Gating strategies for Fig.8B-D.

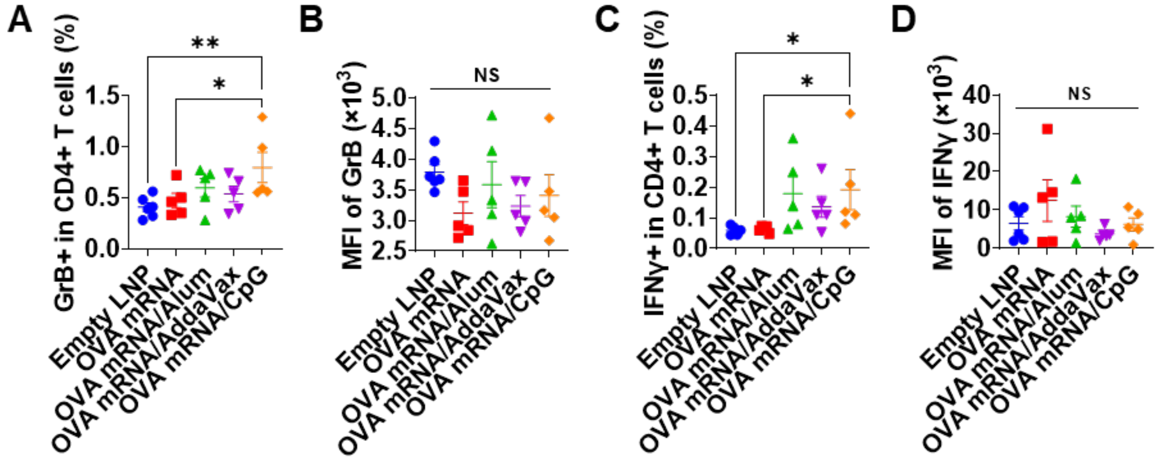

Figure S3. Impact of adjuvants on OVA mRNA-induced CD4+ T cell responses in preventive B16F10-OVA models

Percentages of GrB^+ and $IFN\gamma^+$ cells in CD4+ T cells were shown in A and C, respectively. MFI of GrB and IFNγ in GrB^+CD4^+ and $IFN\gamma^+CD4^+$ T cells were shown in B and D, respectively. One-way ANOVA with Fisher's LSD test was used to compare differences between groups. n=5. *, $p<0.05$; **, $p<0.01$. NS: not significant.

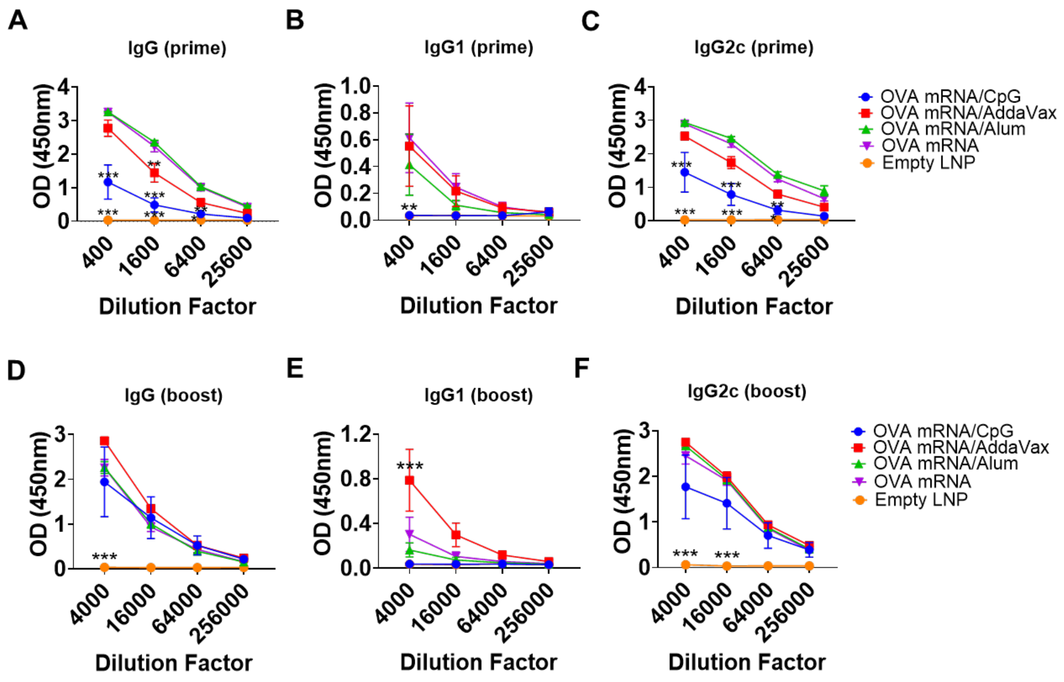

Figure S4. Impact of adjuvants on OVA mRNA-induced antibody responses
Mice were immunized with OVA mRNA alone or in the presence of different adjuvants, or empty LNP. Serum anti-OVA IgG and subtype IgG1 and IgG2c antibody responses were measured 3 weeks after prime (A-C) and boost (D-F). Two-way ANOVA with Dunnett's multiple comparisons test was used to compare OVA mRNA alone with other groups. n=5. *, $p<0.05$; **, $p<0.01$; ***, $p<0.001$.

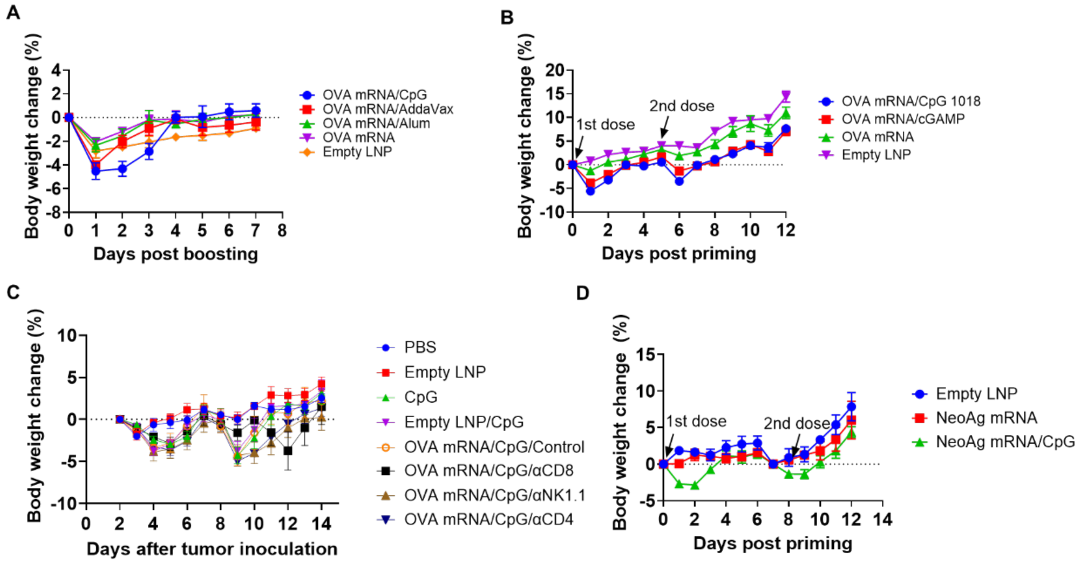

Figure S5. Body weight change in preventive and therapeutic tumor models
A. Body weight change in preventive B16F10-OVA models in Fig.1. B. Body weight change in therapeutic B16F10-OVA models in Fig.2. C. Body weight change in therapeutic B16F10-OVA models in Fig.3. D. Body weight change in therapeutic B16F10 models in Fig.4. To be noted, tumor growth at later timepoints contributed to the weight gain.

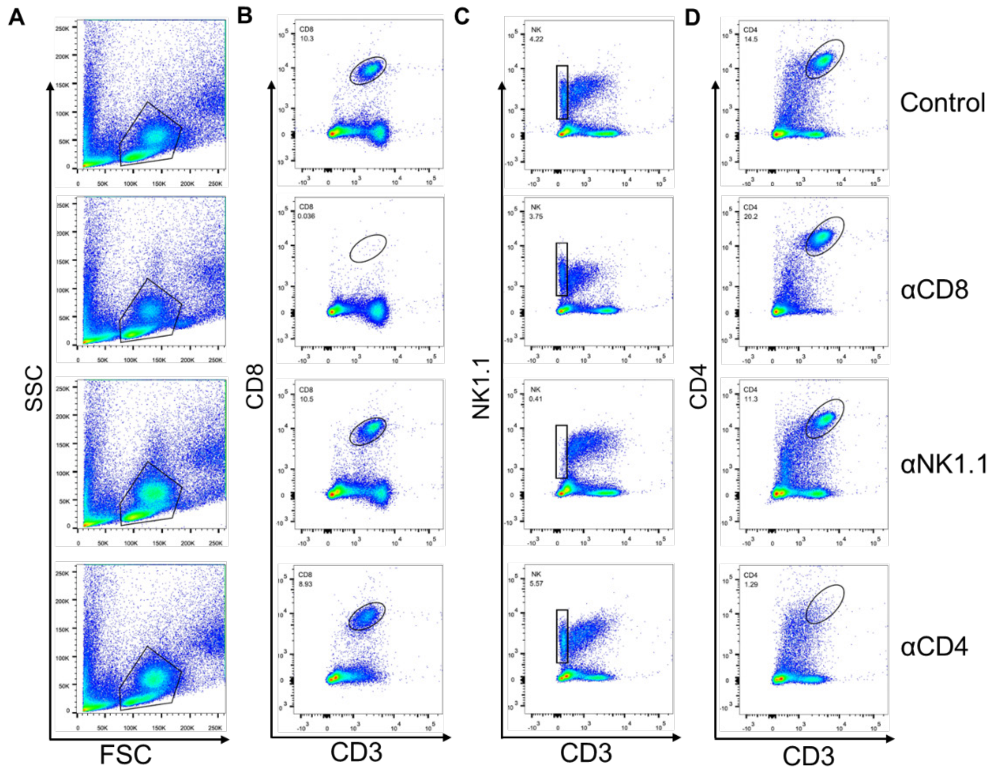

Figure S6. Confirmation of cell depletion in Fig.3

PBMCs were isolated one week after the initiation of antibody-based cell depletion followed by immunostaining and flow cytometry analysis. Cells were first gated based on FSC and SSC in A, then gated based on CD8 and CD3 to identify $CD8^+$ T cells in B, gated on NK1.1 and CD3 to identify NK cells in C, and gated on CD4 and CD3 to identify $CD4^+$ T cells in D. Representative flow cytometry images were shown. Control: isotype control antibody. αCD8: anti-CD8 antibody. αNK1.1: anti-NK1.1 antibody. αCD4: anti-CD4 antibody. Cell depletion was confirmed one week after with similar results.

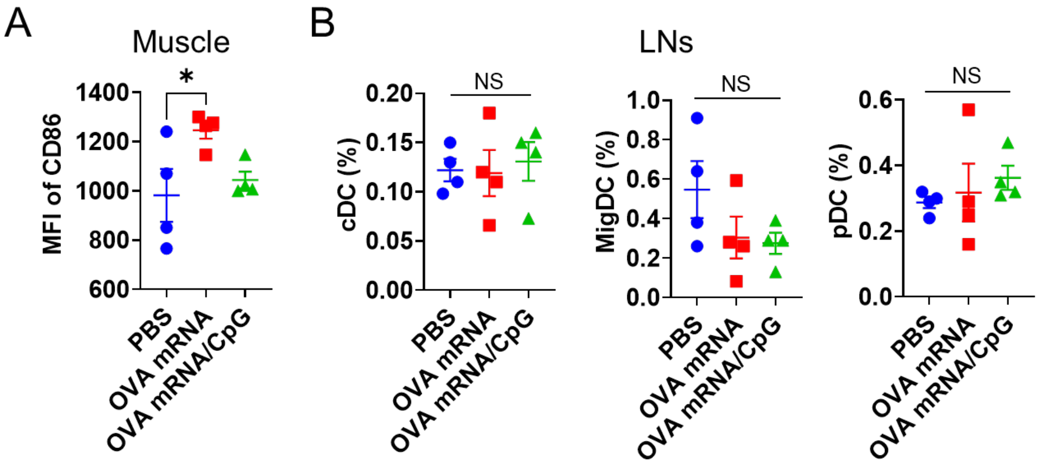

Figure S7. Lack of significant impact of CpG 1018 on CD86 expression in muscle DCs or subtype DC levels in draining LNs

A. Muscle DCs of mice immunized in Fig.6 were analyzed for CD86 expression. B. Draining LNs of mice immunized in Fig.6 were collected and subjected to immunostaining and flow cytometry analysis of percentages of cDC (left), migDC (middle), and pDC (right). One-way ANOVA with Fisher's LSD test was used to compare differences between groups in A-B. n=4. *, p<0.05. NS: not significant.

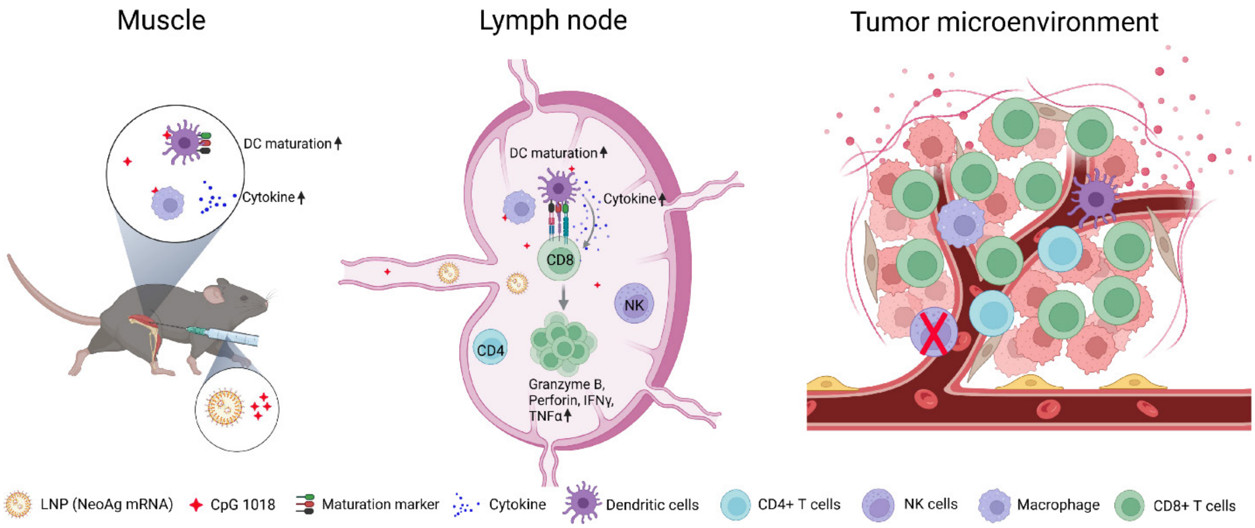

Figure S8. Illustration of functional mechanisms of CpG 1018 in enhancing mRNA vaccine-induced anti-tumor immunity

CpG 1018 stimulates DCs and macrophages to secrete high levels of pro-inflammatory cytokines and also potentiates DC maturation after intramuscular injection together with mRNA vaccines. Similar phenomenon occurs in draining LNs that lead to enhanced antigen-specific $CD8^{+}$ T cell expansion with the ability to secrete high levels of GrB, perforin, IFNγ, and TNFα, leading to significantly enhanced anti-tumor immunity. Inside the tumor, CpG 1018-adjuvanted mRNA vaccination leads to significant increased CD8 to CD4+ T cell ratios, while NK cells show a minimal effect in anti-tumor immunity. Created with BioRender.Com.

Table S1. NeoAg and corresponding WT sequences

	NeoAg	WT
M27	REGVELCPGNKYEMRRHGTTHSLVIHD	REGVELCPGNKYETRRHGTTHSLVIHD
M30	PSKPSFQEFVDWENVSPELNSTDQPFL	PSKPSFQEFVDWEKVSPELNSTDQPFL
M33	DSGSPFPAAVILRDALHMARGLKYLHQ	DSGSPFPAAVILRVALHMARGLKYLHQ
M46	NHSGLVTFQAFIDVMSRETTDTDTADQ	NHSGLVTFQAFIDFMSRETTDTDTADQ
M47	GRGHLLGRLAAIVGKQVLLGRKVVVVR	GRGHLLGRLAAIVAKQVLLGRKVVVVR

(underlined: mutated and corresponding WT amino acids)

Table S2. Accessory sequences

	Peptide sequence
SP	MFVFLVLLPLVSSQCV
Linker #1	GGSGGGGSGG
Linker #2	GGSLGGGGSG
MITD	IVGIVAGLAVLAVVVIGAVVATVMCRRKSSGGKGGSYSQAASSDSAQGSDVSLTA

(Linker #1: after SP and between NeoAgs; Linker #2: before MITD)
